# Where Children Live: Examining Whether Neighborhood Crime and Poverty Is Associated With Overweight and Obesity Among Low-Income Preschool-Aged Primary Care Patients

**DOI:** 10.3389/fped.2018.00433

**Published:** 2019-01-22

**Authors:** Nakiya N. Showell, Jacky M. Jennings, Katherine A. Johnson, Jamie Perin, Rachel L. J. Thornton

**Affiliations:** Division of General Pediatrics and Adolescent Medicine, Johns Hopkins School of Medicine, Baltimore, MD, United States

**Keywords:** obesity, neighborhood crime, neighborhood poverty, primary care pediatrics, intervention

## Abstract

**Introduction:** Low-income and racial/ethnic minority preschoolers (aged 2–5 years) are disproportionately affected by obesity and its associated health consequences. Individual-level factors (e.g., diet) *and* environmental factors (e.g., neighborhood conditions) contribute to these disparities. However, there is limited research examining the role of neighborhood factors on obesity risk specifically among high-risk preschoolers. The objectives of this study are to describe the geographic distribution of preschool patients receiving care at two primary care pediatrics clinics affiliated with an academic medical center, and explore whether exposure to neighborhood crime and poverty is associated with obesity risk among this population.

**Methods:** Cross-sectional multilevel study linking clinical administrative data on patient visits between 2007 and 2012 with data from the American Community Survey and the Baltimore City Police Department. Home addresses of 2–5 year-old patients were geocoded to their neighborhood (i.e., census block group) of residence. We used logistic regression to examine the cross-sectional relationship between obesity and overweight and neighborhood-level factors. All analyses were adjusted for age and gender, and stratified by race/ethnicity (Black, Hispanic, and White).

**Results:** The majority of preschool patients lived in moderate or high crime (84%) or high poverty (54%) neighborhoods. A significantly higher proportion of Black preschoolers lived in high poverty neighborhoods compared to White preschoolers (61% vs. 38%, *p* < 0.001). Among this clinic-based sample of preschoolers, living in high crime or high poverty neighborhoods was not associated with a clinically significant increased odds of overweight or obesity.

**Conclusions:** This study examines the association between neighborhood factors and obesity and overweight among a clinic-based population of low-income racial/ethnic minority preschoolers. The neighborhoods where preschoolers in this sample lived, on average had higher crime counts and poverty than the citywide average for Baltimore. Our findings also suggest that Black preschoolers are exposed to higher levels of neighborhood poverty compared to Whites. While no meaningful association between these neighborhood factors and odds of obesity or overweight was found in this cross-sectional analysis, our findings suggest avenues for future studies informative to the development of clinic-based obesity management interventions aimed at effectively addressing neighborhood contributors to early childhood obesity disparities.

## Introduction

More than 20% of preschoolers (ages 2–5 years) are overweight or obese by the time of school entry (1). Obese preschoolers children are more likely to become obese adults and resultantly are at higher-risk for obesity-related health complications including hypertension, diabetes, and cardiovascular disease (2, 3). While national data suggests obesity rates may be declining among preschoolers, racial/ethnic and socioeconomic (SES) disparities in obesity prevalence in this age group are persistent (4); and threaten to curtail efforts to curb the childhood obesity epidemic.

The mechanisms responsible for producing and sustaining racial/ethnic- and SES-based obesity disparities are likely multifactorial and are hypothesized to include both individual risk factors (e.g., television viewing, increased intake of sugar-sweetened beverage intake) and neighborhood conditions including neighborhood SES, neighborhood safety, and neighborhood crime exposure (5–7).

Neighborhood SES and crime are postulated to influence obesity risk in part through their potential effects on physical activity. Though emerging, data exists supporting an association between these neighborhood conditions and physical activity among children. For instance, a recent systematic review of cohort studies demonstrated that living in unsafe neighborhoods was associated with a reduction in children's (aged ≤ 17 years) physical activity level (8). The majority of studies examined in the aforementioned review incorporated perceived neighborhood safety measures and a lesser number of studies utilized objective measures of crime (8). A prior review conducted by Davison and Lawson, demonstrated that objectively measured local crime rates were inversely associated with children's reported physical activity level (9). Notably, most studies, particularly those focused on preschoolers, have solely utilized subjective measures of neighborhood safety with significant heterogeneity or have examined these types of neighborhood environmental exposures over large geographic areas such as census tracts or counties (9–12).

Despite the potential importance of neighborhood factors such as SES and crime on childhood obesity risk, research examining the association between such factors and obesity risk itself is limited, particularly among low-income racial/ethnic minorities who experience disparities in obesity risk throughout the life course and are also more likely to reside in potentially obesogenic neighborhoods characterized by lower SES and higher levels of crime (6, 8, 13). Fewer studies still have explored the association of neighborhood SES and crime with obesity risk among *clinic-based* pediatric patient populations (14, 15). The findings of such studies can be particularly informative to the development of clinic-based obesity management interventions that integrate strategies responsive to the neighborhood social contexts in which patients live.

In this study, we used data from two urban hospital-affiliated pediatric primary care practices in Baltimore, Maryland to: (1) describe the geographic distribution of residence and neighborhood levels of crime and SES among preschool-aged patients and (2) determine the association of neighborhood levels of crime and SES with the prevalence of obesity and overweight among this clinic-based sample of preschoolers.

## Materials and Methods

### Study Design

This study was a cross-sectional analysis of primary care patient data from preschoolers from October 1, 2007 to November 6, 2012. Patient data were extracted from electronic medical records and manual chart reviews and linked with neighborhood poverty data from the American Community Survey (16) and crime data from the Baltimore City Police Department (BCPD) (17). The Johns Hopkins School of Medicine Institutional Review Board approved this study protocol.

### Setting and Participants

Patient data were from two urban, hospital-based pediatric primary care clinics (Clinic A and Clinic B) affiliated with a large academic medical center in Baltimore, Maryland. Clinic A serves a population that is 65% Hispanic, the majority of whom are children of Mexican and Central American immigrants to the U.S. Clinic B serves a patient population that is approximately 90% non-Hispanic Black. Both sites serve predominantly low-income populations; more than 80% of patients at each site receive health insurance through the state Medicaid program. Preschool-aged patients from both clinic sites were included in this study if they met the following criteria: (1) documented address within Baltimore City limits; (2) ≥1 clinic visit documented in data extraction period with a clear record of height, weight, gender, race/ethnicity and insurer; (3) race/ethnicity documented as White, Black or Hispanic; (4) valid address for geocoding; and (5) biologically valid height, weight and body mass index z-score.

### Data Sources

Patient data were obtained for all study participants from the most recent preventive health visit in each calendar year. The clinic data included the following information from the most recently recorded visit date: date of birth, gender, residential address, zip code, insurance provider/guarantor, language preference, race/ethnicity, patient weight, and patient height.

### Measures

The main outcome measure of overweight and obese was calculated using child age-and sex-specific body mass index (BMI) percentile. We used reported height and weight, and gender, date of birth, and date of visit to calculate the BMI percentile for each patient using standard methodology (18). We report results based on the Centers for Disease Control and Prevention growth chart cutoffs for underweight, normal weight, overweight, and obese. The calculated BMI percentiles were then standardized to produce a BMI z-score for each patient using the 2006 World Health Organization (WHO) Child Growth Standards for children under 60 months of age (19).

The two main neighborhood variables of interest were crime and neighborhood SES. Geographic information system (GIS) software (20) was used to characterize neighborhood crime and neighborhood SES of participants based on geocoded residential addresses. Participant neighborhoods were defined as the 2010 census block group (CBG) using ArcGIS software. Using BCPD data, we operationalized neighborhood crime as the average number of violent crimes (e.g., homicide, aggravated assault) per 1,000 residents in each CBG over the 5-calendar years from 2006 to 2010. Tertiles of neighborhood crime were calculated using the distribution of crime density across the 653 CBGs in the city as the reference. Low-crime neighborhoods had <48 crimes/mi2, moderate-crime neighborhoods had 48–103 crimes/mi2 and high-crime neighborhoods had >103 crimes/mi2. Because Baltimore generally has a high crime rate, and there are not nationally accepted thresholds for determining what constitutes high vs. low crime across all neighborhood contexts, we elected to assess crime as a continuous exposure as opposed to defining high vs. low crime neighborhoods based on the distribution of crime counts across Baltimore CBGs. Using data from the 5-year 2013 American Community Survey (aggregated data from 2008 to 2012) (16), we operationalized neighborhood SES as the percent of households living below the Federal Poverty Line within a CBG. This approach has been used in previous research and is thought to be a more accurate measure of neighborhood poverty level for children as compared to neighborhood median income because it takes into account the number of individuals per household (21). Poverty was dichotomized as high (≥20% of households living below the poverty line) and low (<20% of household living below the poverty line). Thus, the threshold for identifying high poverty or low SES neighborhoods was independent of the median poverty rate across Baltimore City and is based on accepted standard measures.

### Data Analysis

Summary statistics were used to describe the study population by individual and neighborhood level factors overall and by race/ethnicity. We examined potential differences in individual child and neighborhood-level factors by race/ethnicity using Chi-square tests for categorical variables and ANOVA test for continuous variables. Logistic regression was used to evaluate the association between the odds of overweight or obesity and number of violent crimes and separately, neighborhood poverty per CBG. Resulting estimates of associations were calculated for the overall study sample and stratified by race/ethnicity. For neighborhood violent crimes, estimates are reflected as odds ratios for an increase of 10 violent crimes in each child's CBG. For neighborhood SES, estimates are reflected as odds ratios comparing patients living in CBGs with high poverty to patients living in CBGs with low poverty. Statistical significance was defined as an odds ratio that did not cross 1.0 and a *p*-value that was <0.05. All logistic models were adjusted for age and gender.

## Results

### Data Extraction

**Figure 1:**
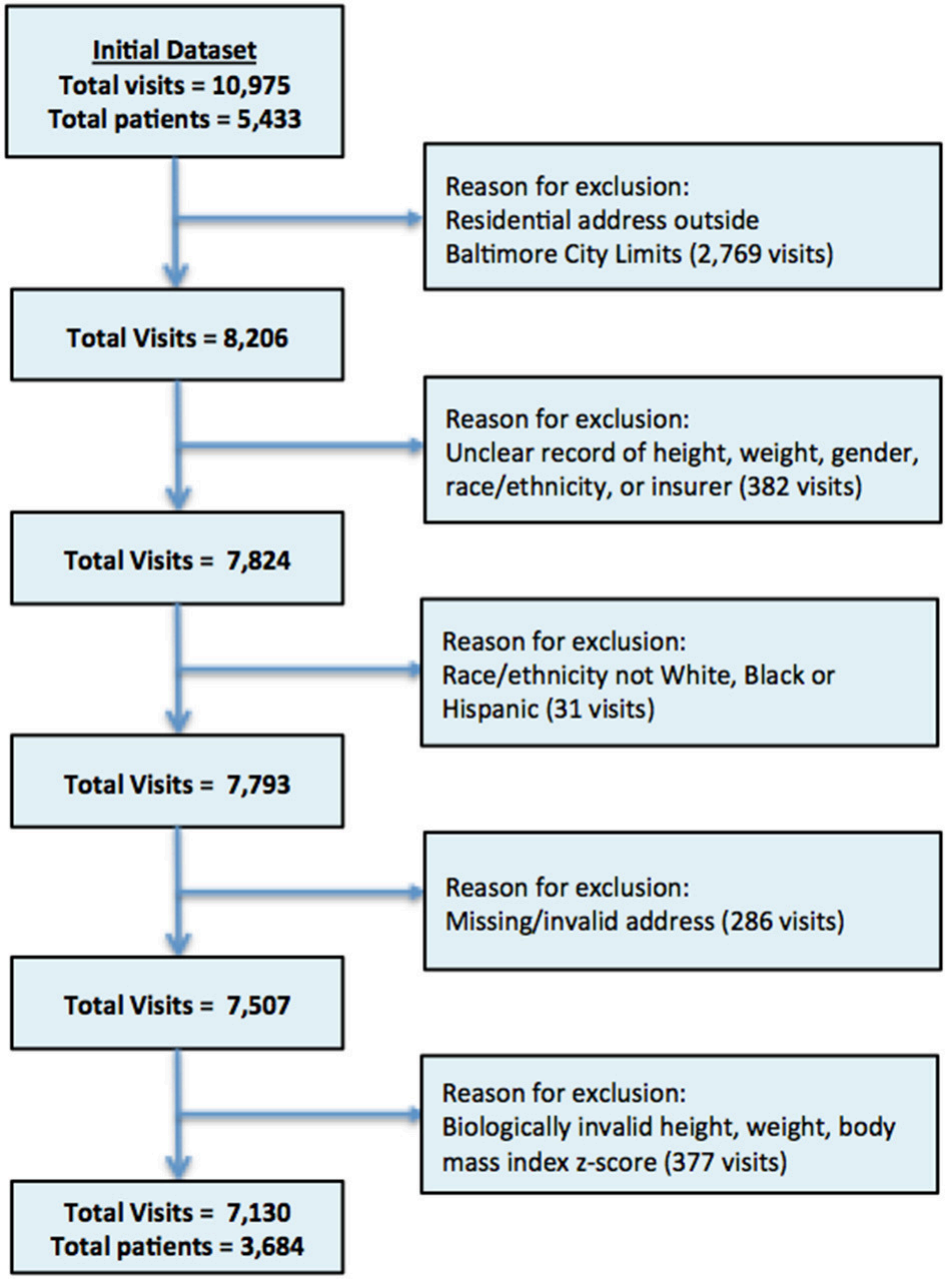
Flow Chart patient data extraction process.

The initial study population included 5,433 unique study participants and 10,975 unique clinic visits (Figure 1). Among these, 2,769 of visits were excluded because the listed residential address was outside the Baltimore City limits. Three hundred and eighty-two of visits with missing or unclear records for height, weight, gender, race/ethnicity, or insurer were also excluded as were children whose race/ethnicity was not listed as White, Black or Hispanic (*n* = 31 visits), children with missing/invalid addresses (*n* = 286 visits) or those where height, weight, or body mass index z-score deemed biologically implausible (*n* = 377 visits). The final study population included 3,684 study participants with 7,130 visits, representing 68 and 65% of initial total patients and total visits, respectively.

### Patient Sample Characteristics and Geographic Distribution

**Figure 2:**
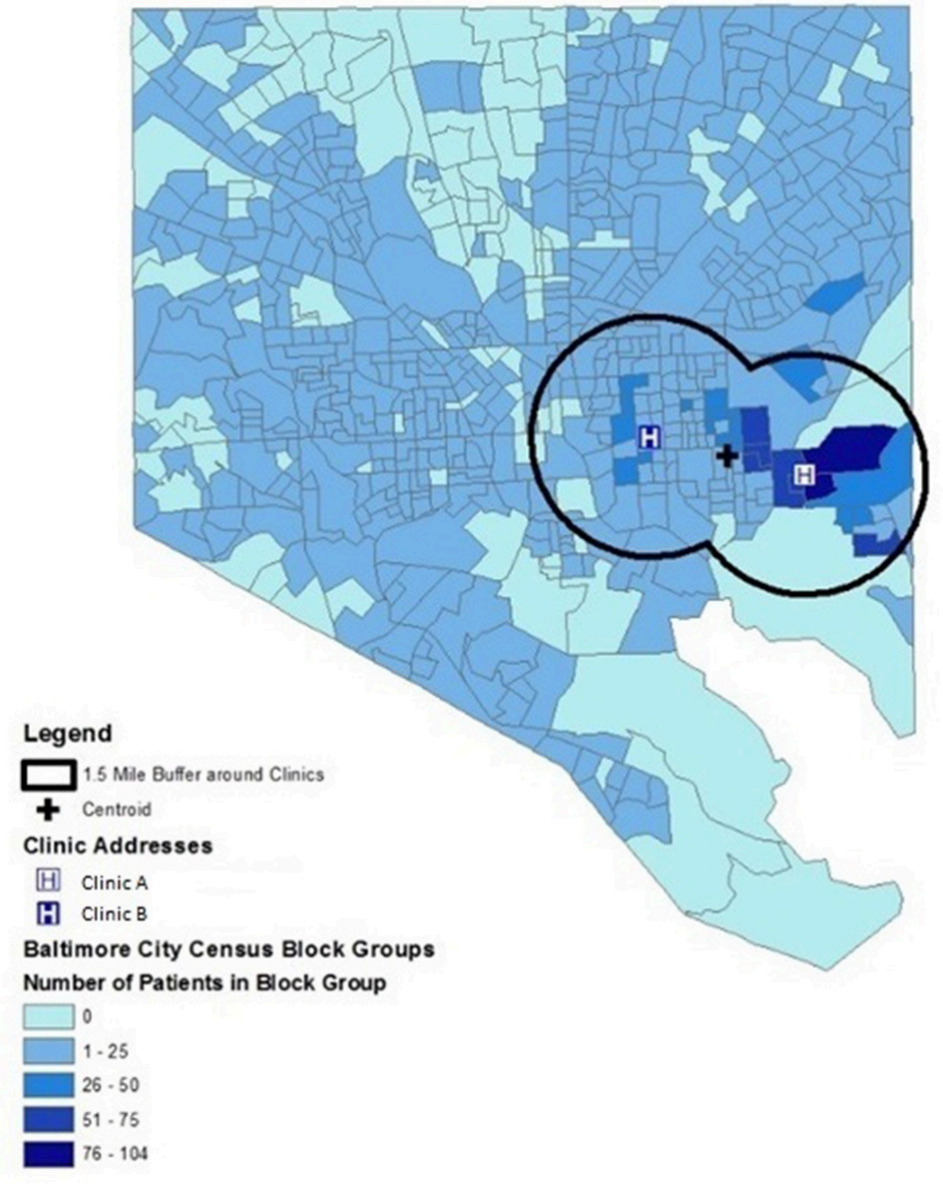
Geographic distribution of neighborhoods of residence for patients residing within Baltimore City limits who received care at two urban, hospital-affiliated primary care clinics. Neighborhoods are depicted as census block groups, and 1.5-mile buffer around each clinic and point location of each clinic are shown.

Figure 2 demonstrates the geographic distribution of neighborhoods of residence for all patients receiving care at Clinic A and Clinic B who live within the Baltimore City limits. This figure demonstrates that the majority of the CBGs with the largest number of clinic patients fall within a1.5-mile buffer of each clinic location. This buffer size was chosen to capture the overlapping area between the two clinic sites. 54.5% (*N* = 2,148) of all patients lived in CBGs that are completely or partially inside the 1.5-mile buffer for the two clinics. The patient population for Clinic A is concentrated within 1.5 miles with 73.9% (*N* = 1,203 patients) residing within this catchment area while only 37.4% (*N* = 865 patients) of clinic B patients reside within 1.5 miles of the clinic. Together, the patients from these two clinics represent 29.1% of all children residing within the catchment area.

The median crime count for the neighborhoods where the study population lived was 75 crimes per CBG as compared to a median crime count of 64 crimes per CBG for Baltimore neighborhoods overall. And, 54% of preschoolers in the study population live in high poverty neighborhoods (≥20% of families living below the federal poverty line) compared to 40% of all Baltimore residents who live in high poverty neighborhoods.

Table 1 provides more detail regarding the demographic characteristics of all patients under study. On average White patients attending these clinics were younger (Whites: mean age 3.8 years vs. Hispanics: mean age 4.0 years, *p* < 0.001), more likely to have private insurance (Whites: 28% vs. Hispanics: 1%, *p* < 0.001), and less likely to be obese as compared to Hispanic patients (Whites: 14% vs. Hispanics: 23%, *p* < 0.001). Black patients were more likely to live in CBGs with high poverty compared to Whites (61 vs. 38%, *p* < 0.001). Similarly, Black and Hispanic patients were both more likely to reside in CBGs with a high number of violent crimes compared to Whites (Blacks: 42%; Hispanics: 44% vs. Whites: 34%, *p* < 0.001).

**Table 1:** Individual and neighborhood characteristics overall and by race/ethnicity among a primary care-based clinic sample of preschoolers, October 1, 2017–November 6, 2012, Baltimore City, Maryland.

	**Overall**	**By race/ethnicity**			p^*^
		**Black**	**Hispanic**	**White**	
*N*	3,684	2,531	904	249	
**CHILD FACTORS**					
Age–mean years (SD)	4.2 (1.2)	4.3 (1.2)	4.0 (1.2)	3.8 (1.2)	<0.001
Source of insurance					<0.001
Public	3,365 (91%)	2,290 (90%)	895 (99%)	180 (72%)	
Private	319 (9%)	241 (10%)	9 (1%)	69 (28%)	
Weight status					<0.001
Below normal	130 (4%)	104 (4%)	15 (2%)	11 (4%)	
Normal	2,363 (64%)	1,677 (66%)	516 (57%)	170 (68%)	
Overweight	589 (16%)	394 (16%)	162 (18%)	33 (13%)	
Obese	602 (16%)	356 (14%)	211 (23%)	35 (14%)	
Gender					0.218
Male	1,858 (50%)	1,301 (51%)	436 (48%)	121 (49%)	
Female	1,826 (50%)	1,230 (49%)	468 (52%)	128 (51%)	
**NEIGHBORHOOD FACTORS**					
CBG more than 20% of HHs in poverty	1,992 (54%)	1,548 (61%)	350 (39%)	94 (38%)	<0.001
CBG number of violent crimes–mean (SD)	105.1 (73.0)	107.0 (71.9)	103.3 (69.8)	93.0 (91.6)	0.010
CBG crime tertile within Baltimore City					<0.001
High crime (>103)	1,542 (42%)	1,059 (42%)	399 (44%)	84 (34%)	
Moderate crime (48–103)	1,550 (42%)	1,112 (44%)	335 (37%)	103 (42%)	
Low crime (less than 48)	591 (16%)	360 (14%)	170 (19%)	61 (25%)	

* *Significance for the association or difference anywhere between race/ethnicity group and given factor, determined by a Chi-square test for categorical factors or ANOVA test for continuous factors*.

### Neighborhood Violent Crime

Table 2 shows estimates of associations between overweight, obesity and number of violent crimes per CBG of patient residence. The odds of obesity or overweight for Hispanic and White preschoolers were not associated with living in a higher crime neighborhood (Hispanics: odds ratio−0.994, 95% CI: 0.974–1.013; Whites: odds ratio−1.008, 95% CI: 0.979–1.037). There was, however, a statistically significant association of the odds of obesity with crime among Black children such that adjusted odds of obesity was 0.978 for every increase of 10 violent crimes in a child's neighborhood (95% CI, 0.959 to 0.998, *p* = 0.031). This result was in the opposite direction of what was hypothesized and was only observed for Black preschoolers. A sensitivity analysis examining whether this association was the result of differences in the neighborhood crime exposure for preschoolers of different races demonstrated that these results were consistent for Black children even after four children living in extremely high crime neighborhoods were excluded (not shown), and when stratifying preschoolers by neighborhood poverty level (not shown).

**Table 2:** Associations between obesity, overweight and overweight or obese and average number of violent crimes in each census block group of patient residence.

		**Black patients**			**Hispanic patients**			**White patients**		
		**Est**.	**95% CI**	***p***	**Est**.	**95% CI**	***p***	**Est**.	**95% CI**	***p***
**CDC-STANDARDIZED BMI PERCENTILE**										
Obese vs. Normal weight	Bivariate	0.979	(0.961, 0.998)	**0.028**	0.985	(0.960, 1.010)	0.238	0.999	(0.962, 1.038)	0.962
	Adjusted^*^	0.978	(0.959, 0.998)	**0.031**	0.987	(0.962, 1.013)	0.330	1.007	(0.969, 1.046)	0.718
Overweight vs. Normal	Bivariate	0.998	(0.983, 1.013)	0.745	1.004	(0.980, 1.027)	0.761	1.013	(0.982, 1.044)	0.424
	Adjusted^*^	0.998	(0.982, 1.014)	0.790	1.003	(0.979, 1.027)	0.836	1.012	(0.981, 1.045)	0.446
Overweight OR Obese	Bivariate	0.990	(0.977, 1.002)	0.108	0.994	(0.974, 1.013)	0.522	1.008	(0.979, 1.037)	0.599
	Adjusted^*^	0.990	(0.976, 1.003)	0.134	0.995	(0.975, 1.015)	0.594	1.011	(0.982, 1.041)	0.469

*Associations are shown as odds ratios for an increase of 10 crimes*.

* *Adjusted for age, gender and proportion of households living under the poverty line in each census block group*.

### Neighborhood Poverty

Table 3 displays estimates of associations between overweight, obesity and neighborhood SES operationalized as neighborhood poverty level dichotomized as high poverty (≥20% of households living below the federal poverty line) and low poverty (<20% of households living below the federal poverty line). Although neighborhood crime and poverty rates are closely related (Pearson correlation estimate 0.23, 95% CI 0.15–0.31, *p* < 0.001), poverty is not associated with obesity or overweight in unadjusted and adjusted analyses in this patient population, overall, and among any of the three racial/ethnic groups studied (Table 3).

**Table 3:** Associations between obesity, overweight and overweight or obese and poverty per CBG of patient residence. Associations are shown as odds ratios comparing those in CBGs with 20% or more of households below poverty line (high poverty) vs. those with less than 20% (low poverty).

		**Black patients**			**Hispanic patients**			**White patients**		
		**Est**.	**95% CI**	***p***	**Est**.	**95% CI**	***p***	**Est**.	**95% CI**	***p***
**CDC-STANDARDIZED BMI**										
Obese vs. Normal weight	Bivariate	0.896	(0.710, 1.131)	0.356	0.989	(0.711, 1.375)	0.947	1.604	(0.771, 3.336)	0.206
	Adjusted^*^	0.884	(0.700, 1.117)	0.302	0.970	(0.695, 1.354)	0.859	1.548	(0.740, 3.236)	0.246
Overweight vs. Normal	Bivariate	1.048	(0.836, 1.314)	0.686	1.113	(0.777, 1.596)	0.559	1.104	(0.514, 2.371)	0.800
	Adjusted^*^	1.039	(0.828, 1.304)	0.742	1.125	(0.784, 1.616)	0.522	1.089	(0.505, 2.348)	0.827
Overweight OR Obese	Bivariate	0.972	(0.815, 1.160)	0.754	1.041	(0.792, 1.369)	0.771	1.341	(0.758, 2.373)	0.314
	Adjusted^*^	0.963	(0.807, 1.149)	0.676	1.042	(0.791, 1.373)	0.768	1.314	(0.740, 2.332)	0.351

* *Adjusted for age and gender*.

## Discussion

In this clinic-based population of 3,684 low-income, predominantly racial/ethnic minority preschoolers, we found that the majority of patients lived in neighborhoods with high levels of crime and poverty. Among this sample of children from predominantly high poverty neighborhoods we found no statistically significant association between neighborhood SES and child overweight or obesity. Furthermore, while we found a statistically significant association between neighborhood crime and obesity among Black preschoolers such that there was a statistically significantly *lower* odds of obesity for every 10 count increase in crimes, this is likely not a clinically significant association nor is it consistent across all racial/ethnic groups within the study sample.

Our findings are in contrast to some other studies that have found statistically significant associations between neighborhood SES and neighborhood crime and child obesity (11, 22–24). Potential explanations for the lack of associations observed in our study include unmeasured neighborhood-level exposures that may contribute to obesity risk such as the neighborhood food environment and proximity to parks or other recreational facilities. Additionally, we were unable to capture additional individual- or family-level contributors to obesity risk among children including *child* diet, sedentary and physical activity behaviors, and *parent* weight status or parent diet and physical activity behaviors. Our analysis further relies on violent crime reports obtained from the Baltimore City Police Department and does not take into account parental perceptions of neighborhood crime or safety.

Our study utilizes a convenience sample of a clinic-based populations serving predominantly low-income Black and Hispanic patients who received primary care, and hence may have limited generalizability to other populations. We found that these preschoolers live in higher crime neighborhoods and in lower SES neighborhoods on average than do residents in Baltimore, Maryland overall. Also, the preschoolers in our sample represent only 29% of all children living in the included CBGs and, thus, may not be representative of all children in the included neighborhoods.

Furthermore, though we assume that the number of violent crimes within a CBG is reflective of a child or family's experience of crime and is relatively stable over time. This may not hold true for all patients. Perceptions or fear of crime may represent a stronger driver of behavioral patterns than objective measures. Additionally, the cross-sectional design of our study may hinder our ability to detect true associations between neighborhood exposures and child overweight and obesity.

Despite these limitations, our study makes an important contribution to the existing literature on neighborhood context and child obesity risk. First, this is one of a few studies to describe the geographic distribution and neighborhood environmental exposures with respect to crime and SES for preschool-aged primary care patients. Secondly, the neighborhood exposures of interest are objectively measured and assessed on a granular scale since we use census block groups as the neighborhood geography of interest, which, in an urban context, is frequently synonymous with an area that is only a few square blocks in size. As such, we measure the neighborhood exposure in the immediate vicinity around a child's neighborhood of residence, which is strength of our methodological approach. We argue that measuring neighborhood exposures in a small geographic area is critically important for understanding the association of SES, crime and obesity risk among preschoolers because, in many cases, the environment immediately surrounding their home most closely reflects their lived experience (25, 26). Other strengths of this study include a robust sample size of over 3,600 participants and use of objectively measured height and weight data obtained from the electronic health record.

Our approach to understanding the catchment areas of the clinics in an attempt to further elucidate the potential impact of neighborhood SES and crime on the health of preschool-aged patients is underutilized in the existing literature. Instead of assuming a certain geographic radius around each clinic as the catchment area, this study describes the distribution of patients and demonstrates that a significant proportion of preschool aged patients from each clinic live within a 1.5-mile radius of each clinic. As such, this study can inform how other clinical practices characterize the patient population they serve using geography and neighborhood exposures to augment what is known about individual patients and families.

## Conclusions

In summary, in this clinic-based sample of preschool-aged patients, we found that the majority of preschoolers lived in neighborhoods characterized by high or moderate crime and high poverty. Furthermore, Black preschoolers lived in higher poverty neighborhoods compared to Whites. While no clinically significant association was found between the odds of overweight or obesity and neighborhood poverty and crime among this sample, findings from this study have implications for future research centered on the development of interventions addressing child obesity disparities.

## Future Implications

Our findings suggest avenues for future research examining the relationship of neighborhood conditions and childhood obesity risk. In particular, future studies using data from clinical populations should include comprehensive data regarding sociodemographic characteristics of patients and families, intermediate behavioral indicators (child-and parent-level) such as diet and physical activity patterns, and other neighborhood-level exposures. Future research should incorporate both perceived *and* objective measures of neighborhood environments and examine associations between neighborhood exposures and growth trajectories over time. Building this body of work is a critical step in designing and implementing effective clinic- and community-based multi-level pediatric obesity management interventions that are responsive to neighborhood conditions where children and families live.

## Ethics Statement

This study was carried out in accordance with the recommendations of name of guidelines, name of committee with written informed consent from all subjects. All subjects gave written informed consent in accordance with the Declaration of Helsinki. The protocol was approved by the Johns Hopkins University School of Medicine Institutional Review Board.

## Author Contributions

NS, JJ, KJ, and RT contributed to conception and design of this study. KJ and JP performed data analyses. NS revised the initial draft of the manuscript and revised all subsequent drafts. All authors critically revised the manuscript, read and approved the submitted version.

### Conflict of Interest Statement

The authors declare that the research was conducted in the absence of any commercial or financial relationships that could be construed as a potential conflict of interest.
